# Primary Orbital Teratoma With Congenital Anophthalmia in a Neonate: A Rare Case With Histopathological and Radiological Correlation

**DOI:** 10.1155/crop/5032089

**Published:** 2025-06-27

**Authors:** Dejan M. Rašić, Dolika D. Vasović, Miroslav Knežević

**Affiliations:** ^1^Eye Hospital, University Clinical Centre of Serbia, Belgrade, Serbia; ^2^Faculty of Medicine, University of Belgrade, Belgrade, Serbia

**Keywords:** anophthalmia, congenital tumor, histopathology, orbital exenteration, primary orbital teratoma

## Abstract

This case report describes a rare instance of primary orbital teratoma with anophthalmia in a neonate. A 6-day-old female presented with a congenital right orbital swelling and absence of visible ocular structures. MRI revealed a large, well-vascularized orbital mass without intracranial extension, accompanied by malformations in the right cerebral hemisphere. Histopathological examination confirmed a benign, mature/mixed teratoma comprising elements from all three germ layers, including neuroectoderm, mesoderm, and endoderm, with no evidence of malignancy. The patient underwent successful orbital exenteration with an implant at 3 weeks of age.

## 1. Introduction

Orbital teratomas are rare congenital tumors accounting for approximately 1% of orbital masses in children. They originate from pluripotent germ cells and can present as primary or secondary tumors, with primary teratomas arising directly within the orbit and secondary ones extending from intracranial or nasopharyngeal regions [1, 2]. Histologically, these tumors contain tissues from all three germ layers and can be classified as mature, immature, or mixed based on cellular differentiation [3, 4]. While most cases are benign, immature teratomas may exhibit malignant potential [5, 6].

Clinically, orbital teratomas manifest at birth as unilateral orbital swelling, often accompanied by anophthalmia or microphthalmia [7]. Imaging and histopathological evaluation are critical for diagnosis and management. Surgical excision remains the treatment of choice to prevent complications such as vision loss, proptosis, and potential malignancy [8, 9]. This report presents a rare case of a congenital primary orbital teratoma with anophthalmia, detailing its clinical presentation, histopathological findings, and surgical management.

## 2. Case Report

A 6-day-old female infant was referred to the University Children's Hospital in Belgrade in February 2019 due to right orbital swelling present since birth. The infant had no other visible anomalies, and her family history was unremarkable. The pregnancy was full term and uneventful, with delivery by cesarean section. At birth, the baby weighed 2390 g, measured 49 cm, and had a head circumference of 32 cm, with an Apgar score of 8. Initial abdominal ultrasonography and laboratory tests were normal. The mother was HIV negative based on standard antenatal screening, and the neonate was not exposed to HIV during pregnancy.

**Figure 1:**
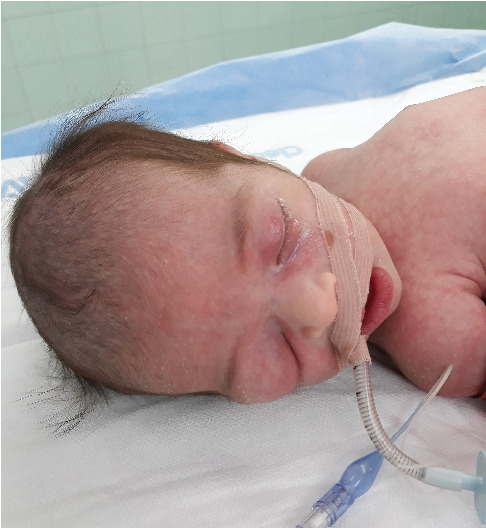
Preoperative photograph of the neonate showing a prominent right orbital swelling with complete absence of visible ocular structures, consistent with congenital anophthalmia. The right eyelids are closed and mildly elevated due to the underlying orbital mass. The patient is intubated and under sedation in preparation for surgical intervention.

Ophthalmological examination revealed a significant right orbital mass. The eyelids were tightly closed, and the structures of the right eyeball were not visible. The left eye was unremarkable (Figure 1).

**Figure 2:**
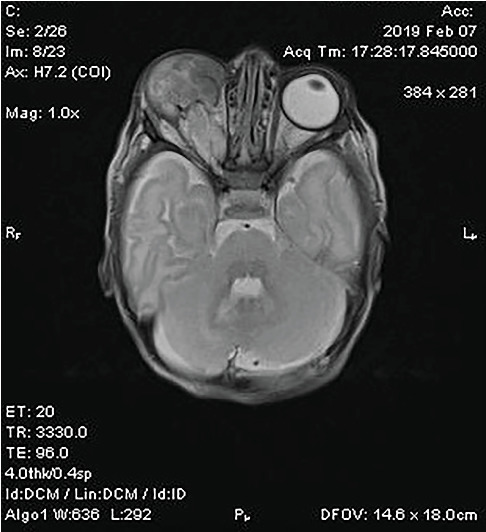
Axial T2-weighted MRI showing a large, heterogeneous, well-circumscribed mass in the right orbit, causing orbital wall remodeling without intracranial extension. The left orbit appears unremarkable.

MRI of the orbit demonstrated a large, heterogeneous, well-vascularized mass in the right orbit measuring 25 × 17 × 33 mm. The mass caused remodeling of the orbital walls but no infiltration. The right optic nerve was fully developed and partially infiltrated, with a slightly thicker optic tract compared to the left side. Structures of the right eyeball were absent. Additional findings included cerebral malformations in the right hemisphere, such as irregular gyration of the occipital lobe, reduced volume of the lower temporal and parahippocampal gyri, focal cortical dysplasia, and partial agenesis of the corpus callosum. No evidence of intracranial tumor extension was observed (Figure 2).

At 3 weeks of age, the patient underwent an excisional biopsy, performed as an eyelid- and conjunctiva-sparing orbital exenteration with orbital implant placement. The postoperative course was uneventful, and no adjuvant therapy was deemed necessary after histopathological confirmation of the diagnosis.

**Figure 3:**
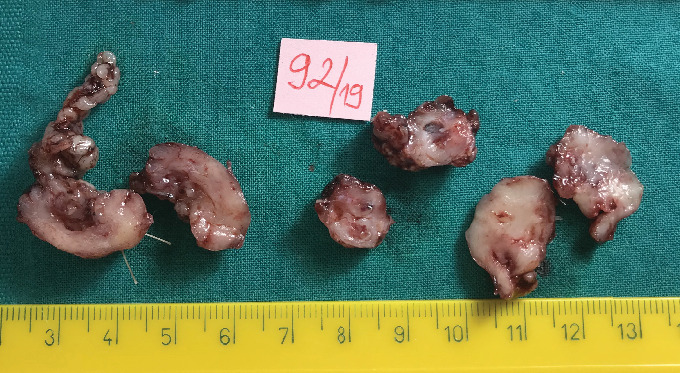
Gross examination of the excised orbital mass showing multiple irregular, gelatinous, whitish specimens, with the largest measuring 25 × 16 × 17 mm.

Gross examination revealed three biopsy specimens, the largest measuring 25 × 16 × 17 mm. The cut surface of the specimens appeared gelatinous and whitish (Figure 3).

**Figure 4:**
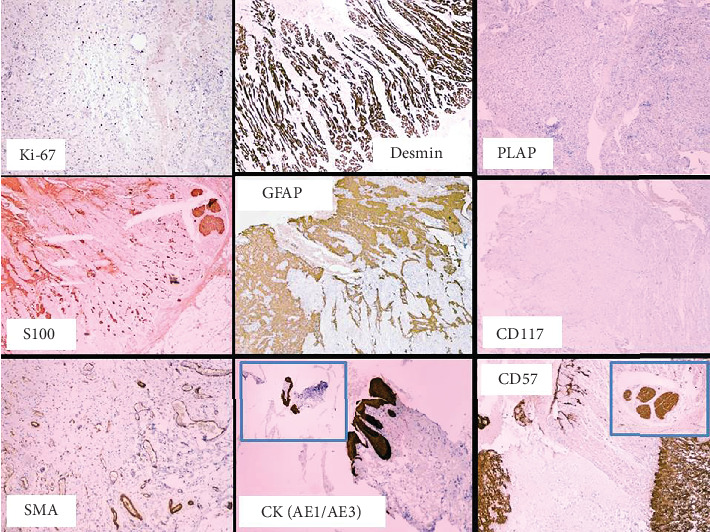
Immunohistochemical analysis of the orbital mass showing staining for various markers: Ki-67 (low proliferative index), desmin (positive), PLAP (negative), S100 (positive), GFAP (positive), CD117 (negative), SMA (positive), CK (AE1/AE3) (positive), and CD57 (positive). These findings confirm the presence of mature elements from all three germ layers, consistent with the diagnosis of a mature teratoma.

**Figure 5:**
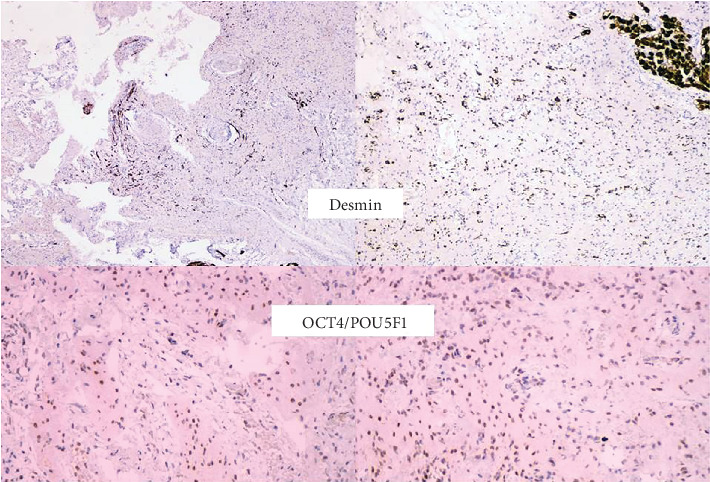
Immunohistochemical staining showing positive desmin expression, indicative of mesodermal differentiation, and OCT4/POU5F1, an embryonic stem cell marker associated with early germ cell development. These findings support the diagnosis of a mature teratoma containing elements from multiple germ layers.

Microscopically, the tumor was not encapsulated and consisted of mature stromal elements derived from all three germ layers: neuroectoderm (brain tissue, nerves, choroid plexus, and ependyma), mesoderm (connective tissue, fat, and striated muscle), and endoderm (gastrointestinal and respiratory epithelium). Immature stromal and glial components were also present, but no cellular atypia or mitotic activity was observed. Ocular structures, surface ectoderm components, or malignant features were not identified (Figures 4 and 5).

Based on the clinical, imaging, and histopathological findings, a diagnosis of congenital, primary orbital, benign, mature/mixed teratoma with anophthalmia and no intracranial extension was established.

## 3. Discussion

Orbital teratomas are rare congenital tumors that present diagnostic and therapeutic challenges due to their complex histopathological features and potential associations with craniofacial anomalies. These tumors arise from misdirected or ectopic primordial germ cells or pluripotent stem cells that differentiate into tissues from all three germ layers: ectoderm, mesoderm, and endoderm [1, 2]. Teratomas are classified as mature, immature, or mixed based on the degree of tissue differentiation and can exhibit a benign or malignant clinical course [3–12].

Mature orbital teratomas, like the one in this case, are typically benign and well-differentiated, comprising a variety of tissue types, such as brain tissue, adipose tissue, and epithelium. Immature teratomas, on the other hand, contain immature neuroepithelial elements and may demonstrate a higher risk for malignancy [3, 5]. The grading of immature teratomas, based on the extent of immature neuroepithelial tissue, aids in determining the prognosis and the need for additional therapeutic interventions [5, 6].

The management of orbital teratomas relies on early and complete surgical excision to preserve function and prevent potential complications, such as compression of adjacent structures or malignant transformation [3, 4, 7, 8, 10]. In this case, the successful orbital exenteration performed at 3 weeks of age resulted in the complete tumor removal without recurrence or the need for adjuvant therapy. Postoperative outcomes were favorable, underscoring the importance of early diagnosis and intervention in such cases.

The immunohistochemical findings in this case demonstrated positive staining for mesodermal markers, such as desmin and SMA, along with the embryonic stem cell marker OCT4/POU5F1, confirming the diagnosis of a mature teratoma. These findings align with previously reported cases, highlighting the role of immunohistochemistry in supporting histopathological evaluation, although it is not diagnostic on its own [2, 6].

To date, only one other case of a mature primary benign orbital teratoma associated with anophthalmia has been reported, in a 7-year-old girl with a history of an orbital mass since birth [4]. The current case adds to the limited literature, emphasizing the rarity of such tumors and the need for meticulous clinical, radiological, and histopathological evaluation to ensure accurate diagnosis and optimal management.

While orbital teratomas have not been directly associated with perinatal HIV exposure, studies suggest that HIV-exposed but uninfected neonates may have altered immune profiles, potentially increasing their vulnerability to various neoplasms. Vertical transmission of HIV and maternal immunosuppression have been linked to higher risks of malignancies in early childhood, including rare tumors [13, 14]. In our case, the mother tested HIV negative during pregnancy, and the neonate was not HIV exposed. Nevertheless, it remains crucial to consider maternal HIV status in the comprehensive evaluation of congenital tumors.

## 4. Conclusion

This case highlights the clinical presentation, imaging characteristics, and histopathological features of a rare primary orbital teratoma with anophthalmia in a neonate. Early surgical excision remains the cornerstone of treatment, yielding favorable outcomes and preventing complications. Comprehensive histopathological analysis, supported by immunohistochemistry, is essential for accurate diagnosis and classification of these rare tumors. This report contributes to the limited body of literature on orbital teratomas and underscores the importance of multidisciplinary management in achieving optimal patient outcomes.

## Data Availability

The data that support the findings of this study are available from the corresponding author upon reasonable request.
